# Combined high expression of CD47 and CD68 is a novel prognostic factor for breast cancer patients

**DOI:** 10.1186/s12935-019-0957-0

**Published:** 2019-09-11

**Authors:** Jingping Yuan, Huihua He, Chuang Chen, Juan Wu, Jie Rao, Honglin Yan

**Affiliations:** 1 Department of Pathology, Renmin Hospital of Wuhan University, No.99, Zhangzhidong Road, Wuchang District, Wuhan, 430060 Hubei People’s Republic of China; 2 Department of Breast and Thyroid Surgery, Renmin Hospital of Wuhan University, Wuhan, 430060 Hubei People’s Republic of China

**Keywords:** Breast cancer, Hormone receptor-negative breast cancer, CD47, CD68

## Abstract

**Background:**

Avoiding the phagocytosis by tumor-associated macrophages (TAMs) is necessary for the growth and metastasis of solid tumors. CD47 binds to the receptor signal-regulatory protein-α (SIRP-α) on the macrophages to avoid normal phagocytosis. In this study, we evaluated the expression and prognostic significance of CD47 and CD68-labeled TAMs in breast cancer solid tumors.

**Methods:**

Two hundred seventeen cases of breast cancer tissues and 40 cases of benign breast lesions were collected for immunohistochemical staining of CD47 and CD68.

**Results:**

Both of the CD47 and CD68 expression were significantly higher in breast cancer tissues (*P* < 0.001), and associated with multiple clinicopathological parameters in breast cancer (*P* < 0.05). However, CD47 or CD68 expression alone was not an independent predictor of poor DFS in multivariate survival analysis (*P* > 0.05). Interestingly, combined high expression of CD47 and CD68 (CD47^high^CD68^high^) not only had a significant association with advanced TNM stage, histological grade, LNM, ER status, PR status and recurrence (*P* < 0.05), but also displayed a poorer 5-DFS (*P* = 0.011). Strikingly, CD47^high^CD68^high^ served as a novel independent prognostic factor for poor DFS compared to the expression of CD47 or CD68 alone (*P* = 0.045). Furthermore, our study also showed for the first time that the prognostic significance of CD47^high^CD68^high^ not only in breast cancer in general, but also in hormone receptor-negative breast cancer in particular.

**Conclusions:**

Combined detection of CD47 and CD68 may provide guidance for the prognosis of breast cancer, especially hormone receptor-negative breast cancer.

## Background

Breast cancer is one of the three most common cancers in the world and is also the most common cancer among Chinese women [1, 2]. China accounts for 12.2% of all newly diagnosed breast cancer cases and 9.6% of all breast cancer deaths worldwide [2]. It is common knowledge that cancer cells have a strong ability to proliferate indefinitely, escape immunity, local invasion and distant metastasis, but the mechanism has not been fully elucidated. Current studies have shown that the development of breast cancer is a complex event involving multiple factors, stages and links. In this process, cancer cells interact with multiple components of the tumor microenvironment to co-evolution [3, 4]. Tumor microenvironment is a complex and comprehensive system, which includes vascular endothelial cells, immune cells, macrophages, fibroblasts, basement membrane, collagen IV and other non-cellular components in addition to tumor cells [3].

Macrophages are the main immune cells infiltrating into the microenvironment of tumors. According to the signal stimulation of the microenvironment, they can polarized into two distinct phenotypes, the classically activated (M1) or the alternative activated (M2) macrophages. Traditionally, M1 macrophages are associated with less tumor invasiveness, while M2 macrophages induce tumor growth and lead to poor prognosis [5]. Tumor-associated macrophages (TAMs), which closely resemble the M2-polarized, are generally characterized by the expression of cell surface marker CD68 [6, 7]. They are important prognostic factors of tumors and have been demonstrated to be correlated with invasion and migration of various tumors [8–10]. Over the past few years, studies on the role of TAMs in the progression of breast cancer have confirmed that TAMs in breast cancer can enhance the invasion of tumor cells and lead to poor prognosis of patients by remodeling extracellular matrix (ECM), inducing angiogenesis, inhibiting the anti-tumor function of cytotoxic T cells, modelling breast cancer cells to escape the host immune system, and recruiting immunosuppressed leukocytes into tumor microenvironment [7].

Recent studies have shown that the reason why macrophages can be counteracted to help the growth of cancer cells is related to the molecular changes on the surface of macrophages and cancer cells [11–13]. Macrophage-mediated programmed cell removal (PrCR) is based on Calreticulin (CRT), an “eat-me signal” on the surface of cancer cells, which interacts with LDL-receptor-related protein (LRP) on the macrophages and recruits macrophages to phagocytize cancer cells [11]. It is an important mechanism for clearing disease and damaged cells before programmed cell death. However, in the course of cancer development, some cancer cells also express another protein, CD47, a kind of “don’t eat me” signal [12]. This protein binds to the receptor signal-regulatory protein-α (SIRP-α) on the phagocytes to inhibit normal phagocytosis [12]. Therefore, the induction of PrCR by “eat-me” signal on tumor cells is counteracted by “don’t eat me” signal, thus avoiding macrophage phagocytosis.

CD47, also known as integrin associated protein (IAP), is a ubiquitous membrane protein belonging to the immunoglobulin superfamily. Compared with normal cells, CD47 is highly expressed in many cancer cells or tissues, such as non-Hodgkin lymphoma cells [14], acute lymphoblastic leukemia cells [15], bladder tumor-initiating cells [16], breast cancer cells [17], myeloma cells [18], hepatocellular carcinoma [19], etc. Recent studies have found that tumor cells evade the phagocytosis and killing effect of macrophages by overexpressing CD47 and induce immune escape of tumor cells [12, 20, 21]. Several studies have shown that the overall survival rate of patients with high expression of CD47 is significantly poorer than that of patients with low expression of CD47 [22–24]. Accordingly, CD47 is considered as a biomarker of several cancer, and its high expression can be used a poor prognostic factor. In breast cancer, only a few studies focused on CD47 at the cytological level, such as breast cancer cell lines, breast cancer stem cells, peripheral blood cells and circulating tumor cells of breast cancer patients [17, 22, 25–27], the expression of CD47 in breast cancer solid tumors have been rarely reported [23].

In this study, we evaluated the expression of CD47 in 217 solid breast cancer tissues and 40 benign breast lesions. Additionally, we measured the expression of TAMs by CD68 immunohistochemical staining. Our study showed for the first time that combined high expression of CD47 and CD68 represented an even better independent predictor for poor prognosis compared to the expression of CD47 or CD68 alone, and could be a novel prognostic factor for breast cancer patients. Moreover, the prognostic significance of combined high expression of CD47 and CD68 not only in breast cancer in general, but also in hormone receptor-negative breast cancer in particular. Therefore, our study provides a new therapeutic target for breast cancer, especially hormone receptor-negative breast cancer.

## Materials and methods

### Tissue samples

The cohort consisted of 217 formalin-fixed paraffin embedded (FFPE) tissue samples of primary invasive breast cancer receiving no treatment prior to surgery. Forty cases of benign breast lesions were collected as controls. All tissue samples were randomly selected from the medical records of the Department of Pathology, Renmin Hospital of Wuhan University between August 2009 and December 2010. Corresponding clinicopathologic data were extracted from medical records and pathology reports. Ages at diagnosis ranged from 29 to 78 years old with an average age of 48.1 years. The main clinicopathological parameters in the study group included age, menopausal status, tumor node metastasis (TNM) stage, histological grade, lymph node metastasis (LNM), estrogen receptor (ER), progesterone receptor (PR), human epidermal growth factor receptor 2 (HER2) and recurrence, as shown in Table 1. Patients were all followed up for 5 years. The follow-up data was defined as the time between the date of the first diagnosis and the date of death or last follow-up time. Written informed consent was obtained from the patients before surgery, and specimens were allowed to be used for scientific research purposes. The study was approved by the Ethics Committee of Renmin Hosptial of Wuhan University.

**Table 1:** Correlation between CD47, CD68 or CD47–CD68 expression and clinicopathological parameters in breast cancer

Clinicopathological parameters	n	CD47			CD68			CD47–CD68		
		High expression (%)	χ^2^	*P*	High expression (%)	χ^2^	*P*	High expression (%)	χ^2^	*P*
Age (years)										
< 50	134	64.2	0.017	0.895	67.9	3.452	0.063	48.5	0.223	0.637
> 50	83	65.1			79.5			51.8		
Menopause										
Before	122	63.9	0.041	0.839	67.2	3.676	0.055	45.9	1.668	0.197
After	95	65.3			78.9			54.7		
TNM stage										
I	13	23.1	11.249	*0.004*	53.8	10.844	*0.004*	7.7	16.769	< *0.001*
II	137	65.0			67.2			46.0		
III	67	71.6			86.6			65.7		
Histological grade										
G1	36	44.4	10.825	*0.004*	69.4	10.500	*0.005*	47.2	11.300	*0.004*
G2	126	64.3			62.9			42.1		
G3	55	78.2			89.1			69.1		
Lymph node metastasis										
No	96	58.3	2.875	0.090	63.5	6.678	*0.010*	40.6	5.759	*0.016*
Yes	121	69.4			79.3			57.0		
ER status										
Negative	121	71.1	5.139	*0.023*	78.5	5.192	*0.023*	61.2	8.253	*0.004*
Positive	96	56.3			64.6			35.4		
PR status										
Negative	120	73.3	9.117	*0.003*	79.2	6.235	*0.013*	55.8	3.948	*0.047*
Positive	97	53.6			63.9			42.3		
HER2 gene										
Non-amplification	163	61.3	2.869	0.090	72.4	0.001	0.981	48.5	0.445	0.505
Amplification	54	74.1			72.2			53.7		
Recurrence										
No	130	31.4	3.957	*0.047*	61.5	7.651	*0.006*	40.8	10.507	*0.001*
Yes	87	72.4			79.3			63.2		

Significant *P*-values are shown in italic type

### Immunohistochemical staining

Immunohistochemical staining of CD47 and CD68 was performed using a standard Envision complex method. Formalin-fixed, paraffin-embedded tissue samples were cut at 4 μm, preheated at 60 °C for 1 h and then deparaffinized and rehydrated endogenous peroxidase activity was blocked by using 3% H_2_O_2_. Antigen retrieval was carried out by microwave heating with citrate buffer (pH 6.0) for 20 min. After that, sections were incubated with primary antibody (anti-CD47, 1:100, ab213079, Abcam, Cambridge, MA, USA; anti-CD68, 1:100, ab213363, Abcam, Cambridge, MA, USA) for 1 h at 37 °C, and then incubated with biotinylated secondary antibody using the Dako Cytomation LSAB2 System-HRP (K0672, DakoCytomation, Carpinteria, CA, USA) for 40 min at 37 °C. After then, the sections were immersed in 3,3′diaminobenzidine (DAB) at room temperature without light for 2 or 3 min. Finally, samples were slight counterstained with hematoxylin for 2 min. The sections with PBS, replacing the primary antibody, were used as negative controls.

### Evaluation of immunohistochemical staining

Immunohistochemical staining of CD47 and CD68 was evaluated by two experienced independent pathologists (Jingping Yuan and Huihua He). The evaluating pathologists were blinded to clinical data. Unclear cases were discussed until consensus was achieved. CD47 protein was mainly expressed in the cytoplasm or cell membrane of tumor cells. We used CD68 as a marker to evaluate TAMs. CD68 protein was predominantly located in cell membranes and cytoplasm of macrophages. Considering the heterogeneity of immunohistochemical staining intensity and distribution, the evaluation of CD47 and CD68 were scored by applying a semi-quantitative immunoreactivity scoring (IRS) system according to the staining intensity and the percentage of positive cells as described by Baccelli et al. [17]. The staining intensity was categorized into four grades as follows, 0 standed for no immunostaining (non-staining), 1 standed for weak (light yellow), 2 standed for moderate (brown yellow), 3 standed for strong (dark brown). The percentage of positive cells was categorized into five grades as follows: 0 standed for none, 1 standed for 1–10%, 2 standed for 11–50%, 3 standed for 51–80% and 4 standed for > 80%. Three most representative felds of high magnification (400×) in each individual case were selected to calculate the final score. Multiplication of the staining intensity and the percentage of positive cells resulted in an IRS ranging from 0 to 12 for each individual case. A case with high expression of protein was scored when scoring between 7 and 12, while a case with low expression of protein was scored when scoring between 0 and 6.

### Statistical analysis

Chi-square test was used to analyze the association between categorical variables. Spearman’s rank correlation analysis was performed to evaluate correlations between variables. The survival curves were disposed by using the Kaplan–Mayer analysis and log-rank test. Prognostic values of variables were estimated by Cox proportional hazard ratio models. Hazard ratios (HRs) and their 95% confidence intervals (CIs) were calculated for multivariate analyses. SPSS software version 19.0 was used to carry out all the statistical analyses and a *P*-value less than 0.05 was considered as statistically significant.

## Results

### Expression and the prognostic value of CD47 in breast cancer

**Fig. 1:**
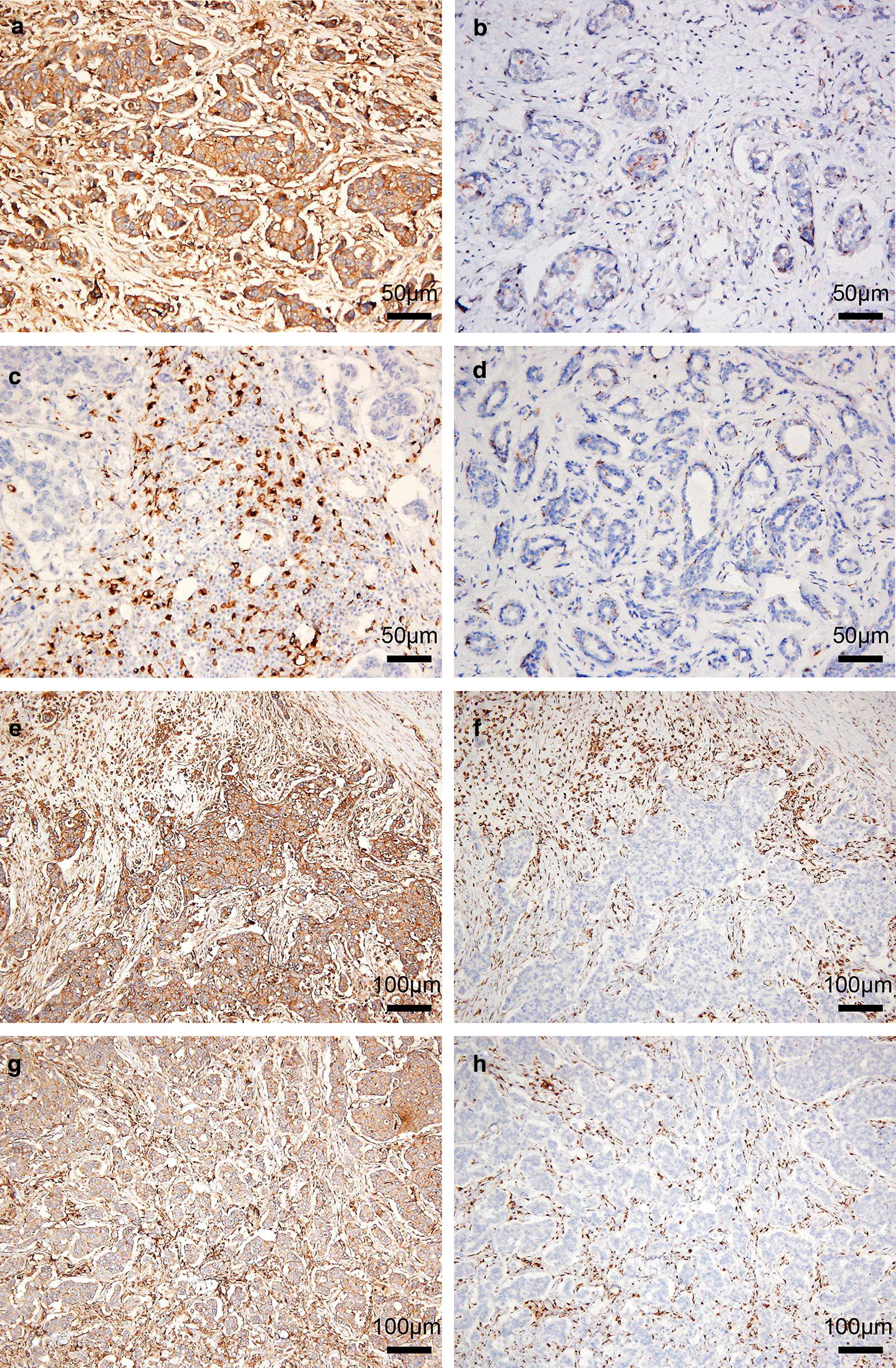
Immunohistochemical staining for CD47 and CD68 in representative tissue specimens. **a** The expression of CD47 in breast cancer tissues. **b** The expression of CD47 in benign breast lesions. **c** The expression of CD68 in breast cancer tissues. **d** The expression of CD68 in benign breast lesions. **e**, **f** Frequent presence of CD68 (**f**) around the nest of breast cancer with high expression of CD47 (**e**). **g**, **h** Frequent presence of CD68 (**h**) in the nest of breast cancer with high expression of CD47 (**g**). 3,3′‑Diaminobenzidine staining (brown), nuclear counterstaining (haematoxylin)

**Fig. 2:**
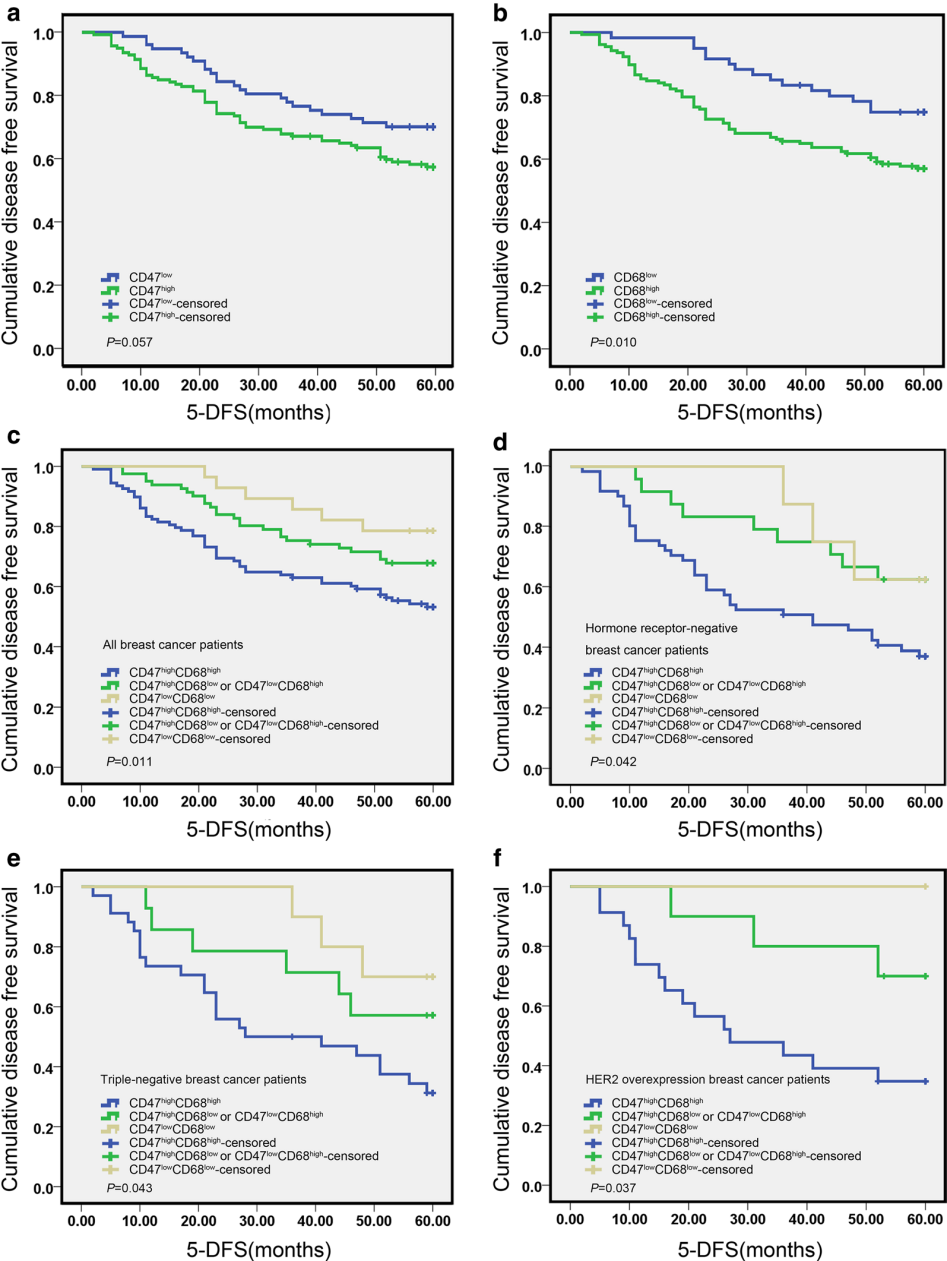
Kaplan–Meier survival analysis showing the correlation between CD47 expression (**a**), CD68 expression (**b**), or CD47-CD68 expression (**c**) and 5-DFS in breast cancer patients (log-rank test). D-F, Kaplan-Meier survival analysis showing the correlation between CD47-CD68 expression and 5-DFS in hormone receptor-negative breast cancer (**d**), TNBC (**e**), and HER2 overexpression breast cancer patients (**f**) (log-rank test)

Two hundred seventeen cases of primary invasive breast cancer and 40 cases of benign breast lesions were collected to examine the expression level of CD47 protein by IHC staining. As shown in Fig. 1a, b, CD47 protein was mainly localized in the cytoplasm or cell membrane of tumor cells in breast cancer tissues, while the immunoreactivity for CD47 in benign breast lesions was negative or weakly positive in the cytoplasm. Of 217 breast cancer tissues, 140 (64.5%) showed high expression of CD47 protein and 77 (35.5%) showed low expression. In 40 cases of benign breast lesions, 12 (30.0%) showed high expression of CD47 protein and 28 (70.0%) showed low expression. The expression of CD47 was significantly higher in breast cancer tissues (χ^2^ = 16.652, *P* < 0.001) as determined by Chi-square test. In addition, the associations between CD47 expression and clinicopathological parameters were further analyzed. As shown in Table 1, high CD47 expression had a significant association with advanced TNM stage (χ^2^ = 11.249, *P* = 0.004), histological grade (χ^2^ = 10.825, *P* = 0.004), ER status (χ^2^ = 5.139, *P* = 0.023), PR status (χ^2^ = 9.117, *P* = 0.003) and recurrence (χ^2^ = 3.957, *P* = 0.047), indicating that CD47 may be one of the prognostic factors of breast cancer. However, Kaplan–Meier analysis and log-rank test showed that high CD47 expression in breast cancer tissues had a limited association with reduced 5-year disease-free survival (5-DFS) (*P *= 0.057) (Fig. 2a). On the other hand, Cox proportional hazards model analysis also revealed that CD47 was not an independent predictor of poor DFS (*P* = 0.063; HR = 1.732, 95% CI 0.970–3.093) as by multivariate analysis including the following co-variables of classical prognostic factors: TNM stage, histological grade, LNM, ER status, PR status and HER2 gene (Table 2).

**Table 2:** Multivariate analysis of prognostic variables including classical prognostic factors and CD47 for disease-free survival in breast cancer patients

Variables	Hazard ratio	95% CI		*P*
		Lower	Upper	
TNM stage (II + I vs. III)	1.852	1.116	3.075	*0.017*
Histological grade (grade 1 + 2 vs. grade 3)	3.661	2.250	5.958	< *0.001*
Lymph node metastasis (no vs. yes)	2.769	1.402	5.472	*0.003*
ER status (negative vs. positive)	0.675	0.369	1.236	0.203
PR status (negative vs. positive)	1.133	0.649	1.978	0.661
HER2 gene (non-amplification vs. amplification)	1.812	1.120	2.932	*0.015*
CD47 (low expression vs. high expression)	1.732	0.970	3.093	0.063

Significant *P*-values are shown in italic type

### Expression and the prognostic value of CD68 in breast cancer

CD68 protein was mainly localized in the membrane and cytoplasm of macrophages (Fig. 1c, d). High expression of CD68 protein was found in 157 cases (72.4%) of breast cancer patients, while in 15 cases (38.4%) of benign breast lesions. It suggested that CD68 was significantly increased in breast cancer tissues in comparison to benign breast lesions (χ^2^ = 18.532, *P* < 0.001). High CD68 expression had a significant association with advanced TNM stage (χ^2^ = 10.844, *P* = 0.004), histological grade (χ^2^ = 10.500, *P* = 0.005), LNM (χ^2^ = 6.678, *P* = 0.010), ER status (χ^2^ = 5.192, *P* = 0.023), PR status (χ^2^ = 6.235, *P* = 0.013) and recurrence (χ^2^ = 7.651, *P* = 0.006). Moreover, the Kaplan–Mayer analysis and log-rank test indicated a strong association of high expression of CD68 with reduced 5-DFS (*P* = 0.010) (Fig. 2b), predicting a poorer survival and lower mortality rate. To further validate the prognostic significance of CD68, Cox proportional hazards model analysis was also performed. As presented in Table 3, CD68 was not an independent predictor of poor DFS (*P* = 0.212; HR = 1.507, 95% CI 0.791–2.873).

**Table 3:** Multivariate analysis of prognostic variables including classical prognostic factors and CD68 for disease-free survival in breast cancer patients

Variables	Hazard ratio	95% CI		*P*
		Lower	Upper	
TNM stage (II + I vs. III)	1.806	1.086	3.004	*0.023*
Histological grade (grade 1 + 2 vs. grade 3)	3.487	2.158	5.636	< *0.001*
Lymph node metastasis (no vs. yes)	2.867	1.455	5.648	*0.002*
ER status (negative vs. positive)	0.627	0.351	1.120	0.115
PR status (negative vs. positive)	1.236	0.713	2.143	0.450
HER2 gene (non-amplification vs. amplification)	1.865	1.148	3.029	*0.012*
CD68 (low expression vs. high expression)	1.507	0.791	2.873	0.212

Significant *P*-values are shown in italic type

### Combined high expression of CD47 and CD68 is associated with poor prognosis in breast cancer

It is reported that CD47 was highly expressed in many cancer cells or tissues, and binded to phagocytes to inhibit normal phagocytosis [12], suggesting that CD47 expression may affect the distribution and expression of macrophages. In accordance with this, we found the frequent presence of CD68-labeled TAMs around or in the nest of breast cancer with high expression of CD47 (Fig. 1e–h). Subsequent analysis of the association between CD47 and CD68 expression showed that the expression of CD68 was positively correlated with that of CD47 (γ = 0.144, *P* = 0.033). In addition, combined high expression of CD47 and CD68 (CD47^high^CD68^high^) were found in 49.8% of breast cancer patients (108/217). What’s more, CD47^high^CD68^high^ had a significant association with advanced TNM stage (χ^2^ = 16.769, *P* < 0.001), histological grade (χ^2^ = 11.300, *P* = 0.004), LNM (χ^2^ = 5.759, *P* = 0.016), ER status (χ^2^ = 8.253, *P* = 0.004), PR status (χ^2^ = 3.948, *P* = 0.047) and recurrence (χ^2^ = 10.507, *P* = 0.001). Strikingly, patients with CD47^high^CD68^high^ displayed a poorer 5-DFS and a worse clinical outcome compared to patients with both low expression of CD47 and CD68 (CD47^low^CD68^low^), or only one protein high expression (CD47^high^CD68^low^ or CD47^low^CD68^high^) (Fig. 2c, *P* = 0.011). Additionally, CD47^high^CD68^high^ served as a novel independent prognostic factor for poor DFS in breast cancer patients as revealed by multivariate analysis subjected to Cox proportional hazards model analysis (Table 4, *P* = 0.045; HR = 1.714, 95% CI 1.012–2.905). These data suggested that the prognostic value of combined high expression of CD47 and CD68 is better than that of CD47 or CD68 alone.

**Table 4:** Multivariate analysis of prognostic variables including classical prognostic factors and CD47–CD68 for disease-free survival in breast cancer patients

Variables	Hazard ratio	95% CI		*P*
		Lower	Upper	
TNM stage (II + I vs. III)	1.804	1.086	2.999	*0.023*
Histological grade (grade 1 + 2 vs. grade 3)	3.368	2.069	5.484	< *0.001*
Lymph node metastasis (no vs. yes)	2.779	1.408	5.485	*0.003*
ER status (negative vs. positive)	0.695	0.378	1.277	0.241
PR status (negative vs. positive)	1.218	0.697	2.128	0.489
HER2 gene (non-amplification vs. amplification)	1.825	1.127	2.955	*0.014*
CD47-CD68 (both negative + one positive vs. both positive)	1.714	1.012	2.905	*0.045*

Significant *P*-values are shown in italic type

### Combined high expression of CD47 and CD68 is associated with the prognosis of hormone receptor-negative breast cancer

**Fig. 3:**
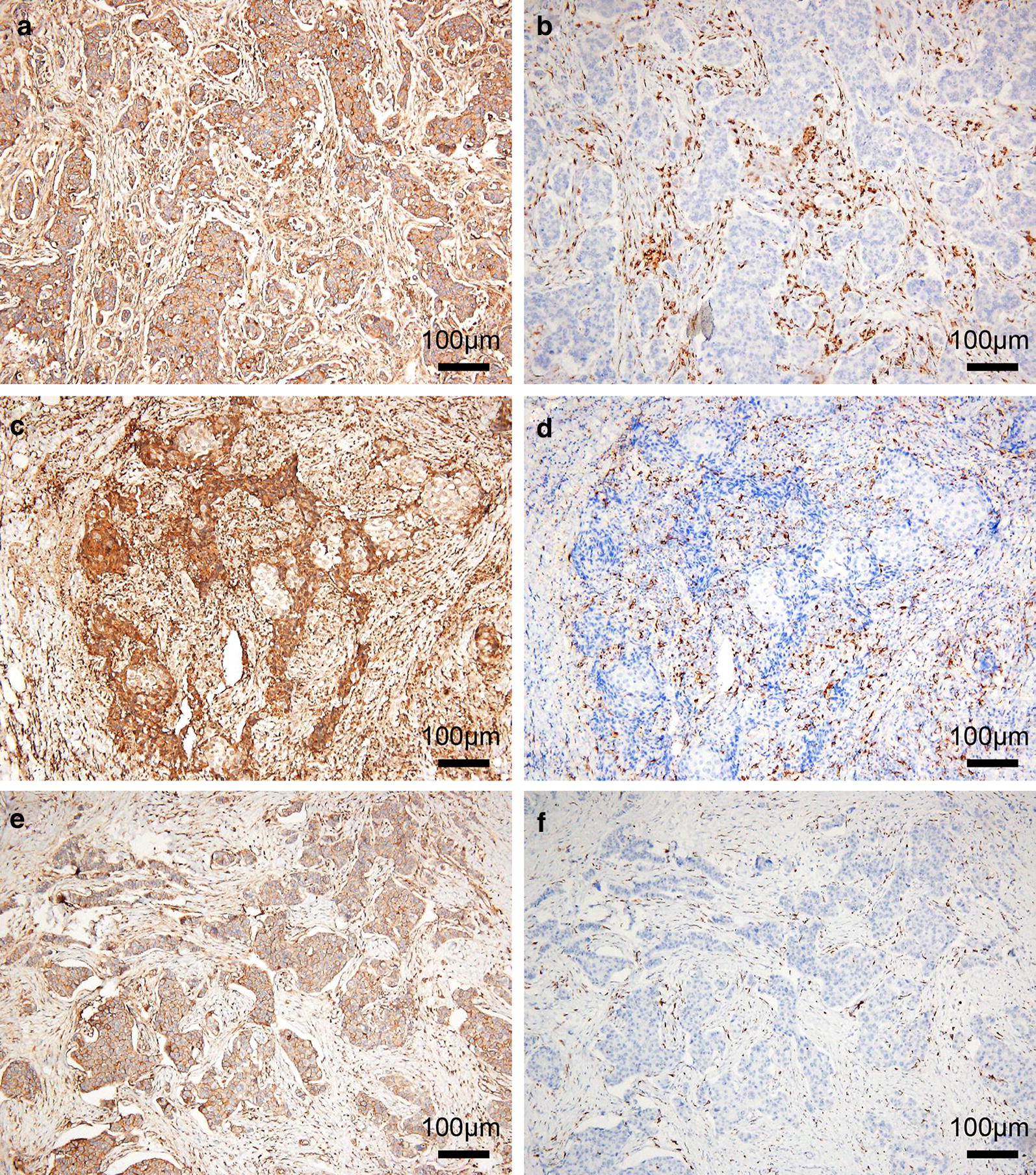
Immunohistochemical staining for CD47 and CD68 in breast cancer with different molecular subtypes. A and B, The expression of CD47 (**a**) and CD68 (**b**) in TNBC. **c**, **d** The expression of CD47 (**c**) and CD68 (**d**) in HER2 overexpression breast cancer. **e**, **f** The expression of CD47 (**e**) and CD68 (**f**) in luminal-type breast cancer. 3,3′‑Diaminobenzidine staining (brown), nuclear counterstaining (haematoxylin)

From Table 1, both of the expression of CD47 and CD68 are significantly correlated with ER and PR status, but not with HER2 gene, suggesting that high expression of CD47 or CD68 may be related to hormone receptors. We also found that CD47 and CD68 expression were more abundant in hormone receptor-negative breast cancer, such as triple-negative breast cancer (TNBC) (Fig. 3a, b) and HER2 overexpression breast cancer (Fig. 3c, d), while the expression density of CD47 and CD68 decreased in luminal-type breast cancer (Fig. 3e, f). Similarly, the incidence of CD47^high^CD68^high^ in hormone receptor-negative breast cancer (65.6%, 61/93) was significantly higher than that in luminal-type breast cancer (37.9%, 47/124) (χ^2^ = 16.297, *P* < 0.001). From Table 5, CD47^high^CD68^high^ had a significant association with advanced TNM stage (χ^2^ = 14.997, *P* = 0.001), histological grade (χ^2^ = 11.714, *P* = 0.003), LNM (χ^2^ = 6.094, *P* = 0.014) and recurrence (χ^2^ = 5.191, *P* = 0.023) in hormone receptor-negative breast cancer patients. Kaplan–Mayer analysis and log-rank test showed that CD47^high^CD68^high^ was associated with reduced 5-DFS (*P *= 0.042) in hormone receptor-negative breast cancer patients (Fig. 2d). Moreover, CD47^high^CD68^high^ was also found to relate to poor 5-DFS in TNBC (*P *= 0.043) and HER2 overexpression breast cancer patients (*P *= 0.037) respectively (Fig. 2e, f). Additionally, cox proportional hazards model analysis indicated CD47^high^CD68^high^ was an independent predictor of poor DFS in hormone receptor-negative breast cancer patients (Table 6, *P* = 0.045; HR = 2.792, 95% CI 1.022–7.626). In summary, combined high expression of CD47 and CD68 is associated with the prognosis of breast cancer, especially for hormone receptor-negative breast cancer.

**Table 5:** Correlation between CD47^high^CD68^high^ and clinicopathological parameters in hormone receptor-negative breast cancer

Clinicopathological factors	n	CD47^high^CD68^high^ (%)	χ^2^	*P*
Age (years)				
< 50	54	64.81	0.034	0.853
> 50	39	66.67		
Menopause				
Before	44	61.36	0.661	0.416
After	49	69.39		
TNM stage				
I	3	33.33	14.997	*0.001*
II	57	52.63		
III	33	90.91		
Histological grade				
G1	7	85.71	11.714	*0.003*
G2	47	48.94		
G3	39	82.05		
Lymph node metastasis				
Yes	54	75.93	6.094	*0.014*
No	39	51.28		
Recurrence				
Yes	50	76.00	5.191	*0.023*
No	43	53.49		

Significant *P*-values are shown in italic type

**Table 6:** Multivariate analysis of prognostic variables including classical prognostic factors and CD47–CD68 for disease-free survival in hormone receptor-negative breast cancer

Variables	Hazard ratio	95% CI		*P*
		Lower	Upper	
TNM stage (II + I vs. III)	1.357	0.681	2.702	0.385
Histological grade (grade 1 + 2 vs. grade 3)	1.839	0.969	3.489	0.062
Lymph node metastasis (no vs. yes)	2.640	1.096	6.356	*0.030*
CD47-CD68 (both negative + one positive vs. both positive)	2.792	1.022	7.626	*0.045*

Significant *P*-values are shown in italic type

## Discussion

CD47 has been considered as a biomarker of several carcinomas, and its high expression is also a poor clinical prognostic factor. However, the exact role of CD47 in the prognosis of breast cancer remains unclear. Manna et al. detected CD47 in breast cancer cell lines, and found that anti-CD47 monoclonal antibody 1F7 could induce the death of four different breast cancer cell lines (MCF-7, HBL-100, MDA-MB-231, and AU-565) [28]. Nagahara et al. found higher expression of CD47 in the bone marrow and peripheral blood of breast cancer patients, moreover, they suggested that CD47 was an important biomarker in breast cancer and functioned as a prognostic factor for DFS [22]. Baccelli et al. showed that MET and CD47 were co-expressed in circulating tumor cells of breast cancer, and the co-expression of MET-CD47 was an independent prognostic factor for survival of luminal-type breast cancer patients [17, 25]. Zhang et al. [29] and Kaur et al. [27] reported that the increase of CD47 in breast cancer stem cells promoted evasion of phagocytosis.

Although these studies suggested that CD47 was overexpressed in breast cancer and associated with the development or prognosis of breast cancer, most studies are based on the detection of CD47 expression at the cytological level, there is little direct evidence to report the expression of CD47 in breast cancer solid tumors and its relationship with prognosis. In this study, 217 cases of primary invasive breast cancer tissues were collected to detect the expression of CD47, and we revealed the direct evidence that CD47 was overexpressed in breast cancer solid tumors. Moreover, high CD47 expression was associated with several clinicopathological parameters including TNM stage, histological grade, ER status, PR status and recurrence. However, high CD47 expression was not an independent and significant prognostic factor for DFS in a multivariate analysis. These results suggest that CD47 may be involved in the progression of breast cancer, but it has a limited prognostic role.

In the microenvironment of tumor inflammation, TAMs infiltrate into tumor masses and suppress the anti-tumor functions of cytotoxic T cells by secreting anti-inflammatory cytokines, they also recruit immunosuppressive leukocytes to the tumor microenvironment [7]. Accumulative evidence suggests that high TAMs density in tumors influences the overall progress of breast cancer and correlates with poor prognosis [7]. Bingle et al. reported that the increase of macrophage density in more than 80% of breast cancer patients was associated with poor prognosis, and patients with higher TAMs density had a significantly worse recurrence-free survival (RFS) and overall survival (OS) [30]. The relationship between the high infiltration rate of TAMs and the signs for poor prognosis such as high grade of tumors, low ER or PR receptor status, and high mitotic activity of tumors has also been reported [31]. In this study, CD68-labeled TAMs was significantly increased in breast cancer, and high macrophage infiltration was associated with advanced TNM stage, histological grade, LNM, ER status, PR status and recurrence. Further survival analysis showed a correlation between high expression of CD68 and reduced 5-DFS, but a limited prognostic value of CD68 expression was observed based on the multivariate Cox proportional analysis.

Although the high expression of CD47 or CD68 had a limited prognostic role value according to the multivariate Cox proportional analysis, our study showed for the first time that combined high expression of CD47 and CD68 represented an even better independent predictor for poor DFS compared to the expression of CD47 or CD68 alone. Why did the patients with CD47^high^CD68^high^ displayed a more significant prognostic effect on the prognosis? We hypothesized that this may be related to immune escape. Since CD47 on the surface of tumor cells binds to the receptor SIRP-α on the macrophages to inhibit normal phagocytosis [12], the anti-phagocytosis effect based on CD47/SIRP-α may be enhanced when CD47 expression is up-regulated and macrophage density is increased, thus affecting the prognosis of breast cancer. Another question is why macrophage aggregation is more frequent in cancer tissues with high CD47 expression? In this study, the expression of CD68 was positively correlated with that of CD47, and CD47^high^CD68^high^ was found in 49.8% of breast cancer patients. Koelzer et al. reported that the prognostic impact of CD68^+^ infiltrates is strongly modified by the expression of the anti-phagocytic molecule CD47 on colorectal tumors [32]. On the other hand, aggregation of macrophages also affects the expression of CD47. Tumor immunosurveillance is a well-established mechanism of tumor growth regulation [33]. Inflammatory environments have been shown to promote the growth of tumors and some unique antigens on the surface of cancer cells could also induce inflammation [34]. In the inflammatory environments, many types of immune cells, including TAMs, infiltrate into tumor masses. Under this strong selective pressure, tumors need to up-regulate the expression of CD47 to avoid immunosurveillance [33].

Our study also confirmed the first time that the prognostic significance of combined high expression of CD47 and CD68 not only in breast cancer in general, but also in hormone receptor-negative breast cancer in particular. Patients with luminal-type breast cancer generally have a good prognosis with a 5-year relative survival between 75 and 86% [35]. However, hormone receptor-negative breast cancer, including TNBC and HER2 overexpression breast cancer, usually has a poor prognosis. It is worth noting that the prognosis of TNBC is worse due to the lack of targeted therapy. We found that CD47^high^CD68^high^ was more abundant in hormone receptor-negative breast cancer compared with that in luminal-type breast cancer. Moreover, CD47^high^CD68^high^ was more significantly associated with the prognosis of hormone receptor-negative breast cancer in particular. Why does CD47^high^CD68^high^ have a higher prognostic value in hormone receptor-negative breast cancer in particular? Historically, breast cancer has not been considered as a typical immunogenic tumor, however, it is well known that this disease is a highly heterogeneous disease [36]. The heterogeneity is reflected in the fact that although the less aggressive breast cancer does not exhibit high immune cell infiltration, it is highly immunogenic for breast cancer lacking hormone receptors [36]. A possible explanation is that TNBC and HER-2 overexpression breast cancer are highly proliferative types of tumors, and the genetic instability of these types of breast cancer causes the exposure of a large number of tumor antigens and promotes the anti-tumor immune response [36, 37]. Under this immune selective pressure, tumor cells need to up-regulate the expression of CD47 and bind to the receptor SIRP-α on phagocytes to inhibit normal phagocytosis and promote immune escape [33], which may be one of the reasons for the high expression of CD47 and CD68 in hormone receptor-negative breast cancer.

It should be acknowledged that there are also some limitations in this study. Firstly, inflammatory environments have been shown to up-regulate the expression of CD47 to avoid immunosurveillance [33]. Studies also showed that microRNAs (such as miR-34a, miR-155, miR-326, miR-141, and miR-133a) and EMT-induced transcription factors (such as SNAI1 or ZEB1) contribute to CD47 regulation [38–41]. However, the exact mechanism of CD47 overexpression in breast cancer solid tumors still unclear, which needs to be evaluated in the future study. Secondly, the prognostic significance of CD47 and CD68 in breast cancer was discussed only at histological level. The exact role of CD47 and CD68 in breast cancer, especially in hormone receptor-negative breast cancer, still needs to be evaluated in follow-up mechanistic investigations. Thirdly, a variety of growth factors and proliferating factors, such as epidermal growth factor (EGF), transforming growth factor (TGF), fibroblast growth factor (FGF), proliferating cell nuclear antigen (PCNA), Ki67, and Cyclin D1, are strongly involved in cell proliferation, differentiation, invasion and migration, thus regulating the prognosis of cancer. Therefore, exploring the relationship between CD47/CD68 protein and proliferation/growth factor is also essential to better demonstrate the role of CD47 and CD68 in the prognosis of breast cancer. Fourthly, this study assessed prognosis through 5-DFS rather than OS. Since DFS is sometimes not linearly related to OS, the influence of CD47 and CD68 expression on OS is still a topic for future research.

## Conclusion

Our study showed for the first time that CD47^high^CD68^high^ represented an even better independent predictor for poor prognosis compared to the expression of CD47 or CD68 alone in breast cancer patients, especially in patients lacking of hormone receptor.

## Data Availability

Not applicable.
